# Design of a randomized controlled double-blind crossover clinical trial to assess the effects of saliva substitutes on bovine enamel and dentin *in situ*

**DOI:** 10.1186/1472-6831-11-13

**Published:** 2011-04-09

**Authors:** Peter Tschoppe, Olivia Wolf, Michael Eichhorn, Peter Martus, Andrej M Kielbassa

**Affiliations:** 1 Department of Operative Dentistry and Periodontology, University School of Dental Medicine, CharitéCentrum 3, Charité - Universitätsmedizin Berlin (Assmannshauser Strasse 4-6), Berlin (14197), Germany; 2 Department of Biometry and Clinical Epidemiology, CharitéCentrum 4; Charité - Universitätsmedizin Berlin (Charitéplatz 1), Berlin (10098), Germany

## Abstract

**Background:**

Hyposalivation is caused by various syndromes, diabetes, drugs, inflammation, infection, or radiotherapy of the salivary glands. Patients with hyposalivation often show an increased caries incidence. Moreover, hyposalivation is frequently accompanied by oral discomfort and impaired oral functions, and saliva substitutes are widely used to alleviate oral symptoms. However, preference of saliva substitutes due to taste, handling, and relief of oral symptoms has been discussed controversially. Some of the marketed products have shown demineralizing effects on dental hard tissues *in vitro*. This demineralizing potential is attributed to the undersaturation with respect to calcium phosphates. Therefore, it is important to modify the mineralizing potential of saliva substitutes to prevent carious lesions. Thus, the aim of the present study was to evaluate the effects of a possible remineralizing saliva substitute (SN; modified Saliva natura) compared to a demineralizing one (G; Glandosane) on mineral parameters of sound bovine dentin and enamel as well as on artificially demineralized enamel specimens *in situ*. Moreover, oral well-being after use of each saliva substitute was recorded.

**Methods/Design:**

Using a randomized, double-blind, crossover, phase II/III *in situ *trial, volunteers with hyposalivation utilize removable dentures containing bovine specimens during the experimental period. The volunteers are divided into two groups, and are required to apply both saliva substitutes for seven weeks each. After both test periods, differences in mineral loss and lesion depth between values before and after exposure are evaluated based on microradiographs. The oral well-being of the volunteers before and after therapy is determined using questionnaires. With respect to the microradiographic analysis, equal mineral losses and lesion depths of enamel and dentin specimens during treatment with SN and G, and no differences in patients' experienced oral comfort after SN compared to G usage are expected (H_0_).

**Discussion:**

Up to now, 14 patients have been included in the study, and no reasons for early termination of the trial have been identified. The design seems suitable for determining the effects of saliva substitutes on dental hard tissues *in situ*, and should provide detailed information on the oral well-being after use of different saliva substitutes in patients with hyposalivation.

**Trial registration:**

**ClinicalTrials.gov ID. **NCT01165970

## Background

Saliva is well recognized as an important factor in the maintenance of oral health [1]. Hyposalivation is associated not only with Sjögren's syndrome or salivary gland hypofunction in elderly patients, but also with the use of medications that contain antimuscarinic drugs, chemoradiotherapy for head and neck carcinomas, and psychiatric disorders [2,3]. Moreover, various diseases such as Riley-Day, Plummer-Vinson, and Heerfordt's disease can lead to hyposalivation [4-6]. Hyposalivation can significantly increase the incidence of dental caries [7], and might compromise the mucosal integrity, thus resulting in oral pain [8]. Additionally, salivary dysfunction may result in a considerably worse morbidity, sleep disturbances, difficulties in chewing and swallowing, speech problems, loss of taste, and an increased incidence of mucosal infections [9,10]. These adverse effects can lead to predispositions of severe oral diseases and nutritional deficiencies, and may result in an overall decline in quality of life [11,12].

In particular, "radiation caries" (a non-appropriate, but widely spread term), a rapidly developing and highly destructive form of tooth decay, is a well-known consequence of radiotherapy [7]. Radiation-induced hyposalivation is caused by functional changes in the salivary glands. The most severe and irreversible forms of salivary gland hypofunction result from damage to the salivary acinar cells [13]. As secretion rates decrease, saliva becomes more viscous [14], along with a more acidic pH value [15]. These changes compromise the preventive functions of saliva, and lead to a greater incidence of caries after radiotherapy in the head and neck area [9].

Artificial saliva has been shown to be efficacious in relieving the subjective symptoms of hyposalivation [16], and therefore, represents a well-accepted and important treatment option. Several saliva substitutes, which differ with respect to chemical compositions, thickeners or viscosities, have been developed to lubricate and moisten the oral mucosa. A previous study concluded that artificial saliva containing mucin proved to be of benefit to patients suffering from hyposalivation compared to a carboxymethylcellulose (CMC)-based saliva substitute due to a better improvement of the oral functioning, a longer retention time on oral mucosae resulting in a lower amount of application needed per day [17]. However, preference of different saliva substitutes by various groups of patients has been discussed controversially [16,18-21]. Up to now, clinical effects of the polysaccharide-based saliva substitute Saliva natura (SN) have not been documented. It might be speculated that SN alleviates the orals symptoms of hyposalivation better compared to the CMC-based solution due to a similar lubrication of the oral mucosa as mucins. In consideration of the comparative pH of SN and Glandosane (G), the stimulating effect on resting salivation should be nearly the same. Furthermore, compared to the consumption of water or tea, the primary role of these substitutes is to provide a prolonged moisturization of the oral mucosa [7,22,23]. This property should not be accompanied by any negative effects on dental hard tissues (*i.e*., demineralization or erosion) or oral health during the frequent use of saliva substitutes.

Previous *in vitro *studies revealed that some commercially available saliva substitutes possess demineralizing properties on enamel [24,25] and dentin [26]. Glandosane, a commercially available and widely spread saliva substitute, is based on CMC, and has shown detrimental demineralizing effects on enamel and dentin *in vitro *[24-26]. In contrast, enhanced remineralization could be observed after the addition of calcium, phosphates, and fluorides to saliva substitutes. A modified version of Saliva natura (a polysaccharide-based saliva substitute) that is supersaturated with respect to octacalcium phosphate (S_OCP _= 2) was capable of inducing enamel and dentin remineralization *in vitro *[27,28]. However, hitherto no clinical study has confirmed the numerous *in vitro *results showing that saliva substitutes have de- or remineralizing effects on dental hard tissues. Therefore, in the current clinical trial, it is aimed to prove that the use of SN is superior to G with respect to the mineralizing effects on dental hard tissues and that the patients experience a better oral comfort while using SN compared to G. Correspondingly, the null hypotheses are "no difference" between G and SN with respect to mineral parameters and that there are "no differences" in oral comfort while using SN compared to G.

## Methods/Design

### Experimental design and study population

**Figure 1:**
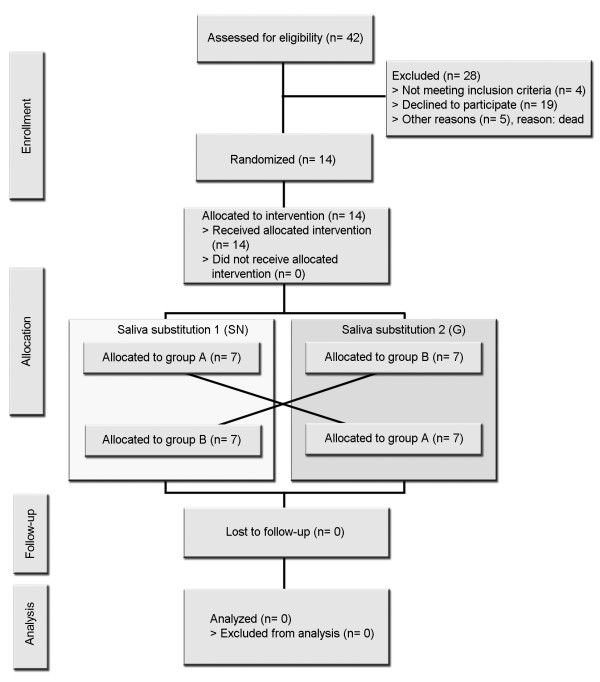
**Study flow chart with presently ascertained patients' figures**.

**Figure 2:**
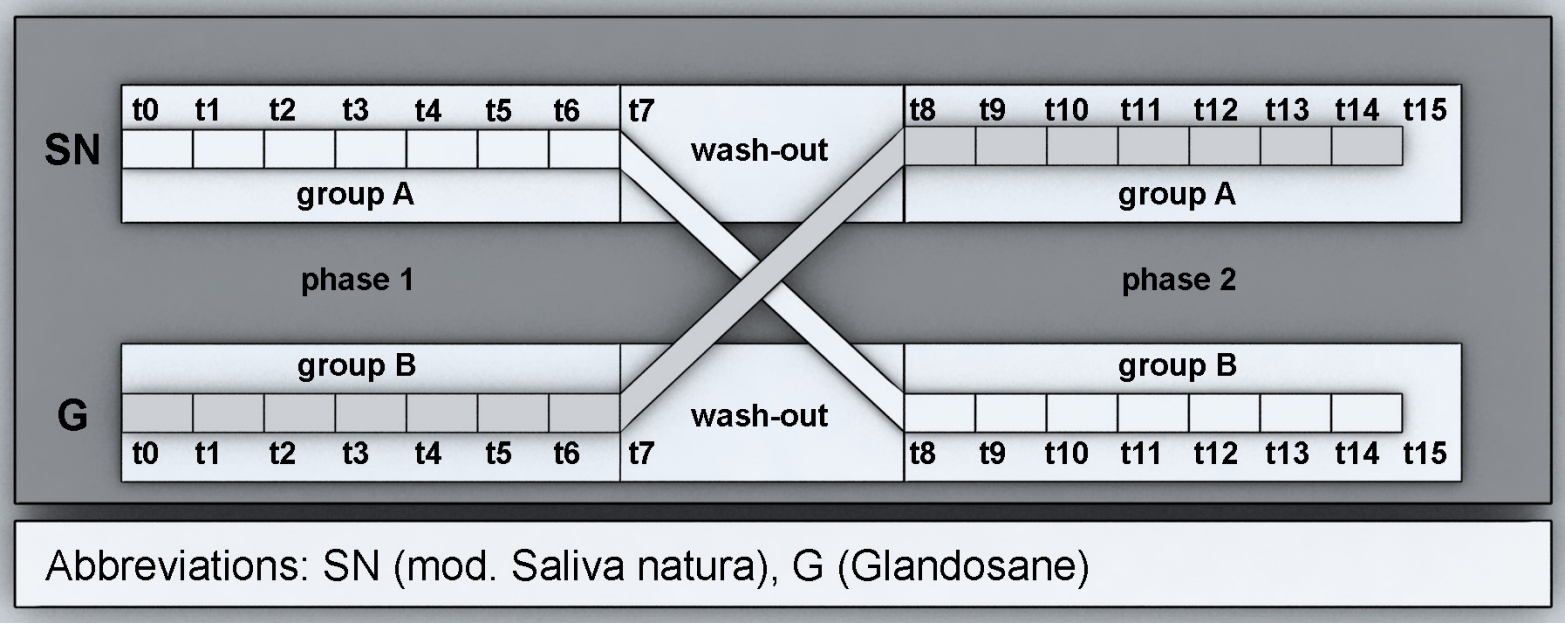
**Study design**. t0: - Integration of specimens into removable (partial) dentures Covering of reference areas/sound surfaces of specimens - Issue of study medication (blinded) - Anamnesis and oral findings - Measurement of unstimulated saliva flow rate - First questionnaire. t5: - Covering of effect areas after five weeks - Measurement of unstimulated whole saliva. t7: - Removal of specimens from the dentures - Measurement of unstimulated saliva flowrate - Second questionnaire - Collecting of spray bottles. t8: - Integration of specimens into the removable (partial) dentures - Covering of the reference areas/sound surfaces of specimens - Issue of study medication (blinded) - Anamnesis and oral findings - Measurement of unstimulated saliva flowrate - Third questionnaire. t13: - Covering of effect fields after five weeks - Measurement of unstimulated saliva flowrate. t15: - Removal of specimens from dentures; repair of dentures - Measurement of unstimulated saliva flowrate - Last questionnaire - Collecting of spray bottles

This study is performed at the Department of Operative Dentistry and Periodontology, University School of Dental Medicine, CharitéCentrum 3, Charité - Universitätsmedizin Berlin, Germany. A controlled, randomized, crossover, double-blind, phase II/III *in situ *trial design is conducted with volunteers who are residents of Berlin. All participants are clinically evaluated by a detailed dental examination and an unstimulated salivary flow rate measurement. Before participating in the study, volunteers are informed concerning the objectives, benefits, and possible risks involved in the study. The research team provides an informed consent form that includes the study details, such as purpose, duration, required procedures, possible advantages, and key contacts. The main inclusion criterion (Table 1) is an unstimulated whole saliva flow of ≤ 0.1 ml/min. Furthermore, one removable dental prosthesis (upper or lower denture) and an age over 18 years are required. Panelists are accepted for study participation only after signing the informed consent forms. The trial period is divided into two phases, and each phase consists of seven weeks with a washout period of seven days between the phases (Figure 1). The volunteers are randomly allocated into two groups. In each study leg, the participants are submitted to treatment with one of the two saliva substitutes (Figures 1 and 2).

**Table 1:** Inclusion and exclusion criteria

Inclusion criteria	Exclusion criteria
• Patients with oncologic diseases of the head and neck area, and implemented radiotherapy of the head and neck area • Men and women at the age of at least 18 years • Unstimulated saliva flow rate < 0.1 ml/ min • Patient's education and written approval of the participation before enrollment • Good prognosis of life expectancy • No paraben allergy • Willingness to co-operate (compliance) of the patient • Ability and willingness to return for follow-up visits • No participation in other study (3 months before and) during the participation	• Pregnancy and lactation period • HIV-infection • Hepatitis B/C virus infection • Reluctance for the storage and disclosure of personal disease data in the context of the study plan • No written approval • Paraben allergy • Concurrent participation in another clinical study • Persons who are accommodated on account of official or judicial arrangement in an institution (§40 Abs.1 S.3 Nr.4 AMG) • Patients who do not wear their dentures at night • Known hypersensitivity to ingredients of Saliva natura and Glandosane (sodium benzoate may cause slight irritation to skin, eyes, and mucous membranes)

The randomization process was performed externally by the Department of Biometry and Clinical Epidemiology (CharitéCentrum 4, Berlin, Germany) using a computer-generated random table (Microsoft Excel, Unterschleißheim, Germany). The investigators were not involved in the randomization process, nor were they aware of the assigned group in any of the outcome evaluations. The identification codes for each panelist were printed on the bottle labels at Charité's pharmacy.

**Figure 3:**
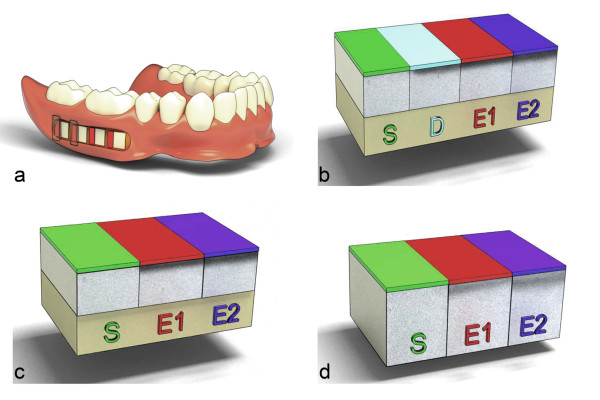
**Arrangement of specimens in the prosthesis and various test fields of both hard tissues**. a: - Lower removable full denture with inserted specimens (mesial two dentin specimens, distal two enamel specimens). b: - Arrangement of artificially demineralized enamel specimens (S: sound surface as reference; D: artificially demineralized surface as reference; E1: effect on the artificially demineralized surface after five weeks; E2: effect on the artificially demineralized surface after seven weeks). c: - Arrangement of sound enamel specimens (E1: effect on sound surface after five weeks; E2: effect after seven weeks). d: - Arrangement of sound dentin specimens (E1: effect on sound surfaces after five weeks; E2: effect after seven weeks)

**Figure 4:**
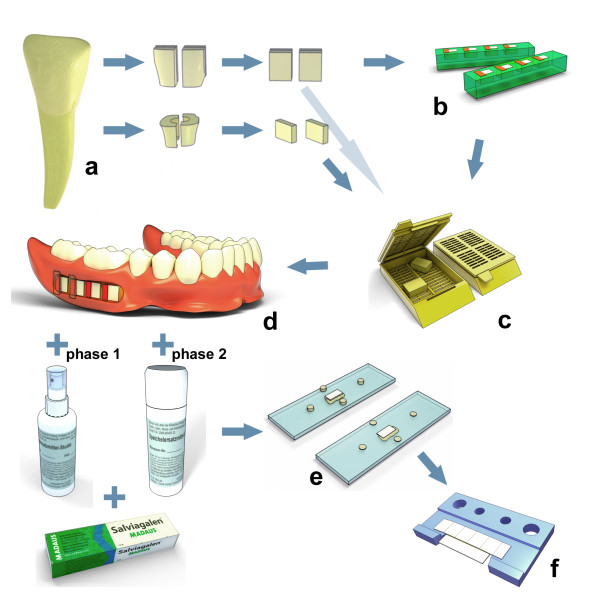
**Study procedure and experimental set-up**. a: - Specimen preparation from permanent bovine incisors - Enamel and dentin surfaces ground flat and polished. b: - Artificial lesion creation in half of the enamel specimens. c: - Sterilization (ethylene oxide) of all specimens (enamel and dentin). d: - Insertion of four specimens into removable dentures during each phase (2 enamel specimens and 2 dentin specimens) - Blinded medication and fluoride-free toothpaste. e: - Preparation of thin sections (100 μm). f: - Microradiographic analysis of thin sections and statistical analysis

A dental study nurse is commissioned to allocate the sealed envelopes, which contains the blinded saliva substitutes from the pharmacy. This procedure guarantees a continuous medication blinding process for both panelists and investigators. After each testing phase, all of the released bottles are collected by the clinical investigators to determine consumption based on weight. The volunteers wear removable dentures comprising four inserted specimens during each study phase (2 enamel and 2 dentin specimens) (Figures 3 and 4d). All of the specimens are obtained from one bovine tooth. Furthermore, the enamel specimens are divided into two subgroups, including sound and artificially demineralized (subsurface lesion) enamel (Figures 3b, c and 4).

The patients are asked to perform individual oral hygiene procedures using a fluoride-free toothpaste (Salviagalen; Madaus, Cologne, Germany). In addition, they are instructed to refrain from using any anti-caries or anti-bacterial agents in addition to their oral hygiene procedures which are continued on a regular basis. Moreover, the participants are advised to avoid highly fluoridated salts and foods.

### Ethical considerations

The trial was approved by the Ethical Committee of Berlin (Office State of Health and Social Affairs, LaGeSo, Berlin, Germany) and by the German Federal Institute for Drugs and Medical Devices (BfArM, Bonn, Germany), and written consent was obtained from each patient according to the current revision of the Helsinki Declaration (originally adopted in June 1964, sixth revision in 2008). EudraCT (European Union Drug Regulating Authorities Clinical Trials) number 2008-005451-23 was assigned to the study by the European Medicines Agency (EMEA, London, United Kingdom). The study is registered under Clinical Trials database (Trial registration: ClinicalTrials.gov ID. NCT01165970). According to valid German law, Glandosane is classified as a pharmaceutical drug, whereas Saliva natura represents a medical device. Since both products are used, the present study is considered a drug study.

### Preparation of artificial salivas

Saliva natura (SN; medac, Germany) was modified by the addition of calcium, phosphates, and fluorides (Table 2), which resulted in a solution with an octacalcium phosphate saturation (S_OCP_) of 2.0 at a buffered pH of 5.95 [27,28]. Glandosane (G; cell pharm, Hanover, Germany), which represents a demineralizing saliva substitute, was tested in its pure form (original drug).

**Table 2:** Composition of the study medication and calculated saturations with respect to calcium phosphates and CaF_2_

	calculated saturation of an aqueous solution with respect to:				pH	ion concentrations (mM)						
**solution**	**DCPD**	**OCP**	**HA**	**CaF_2_**		**Ca**	**PO**_**4**_	**F**	**K**	**Cl**	**Na**	**Mg**

Glandosane	0.2	**0.3**	0.9	--	5.3	1.0	2.6	0	19.0	33.8	14.8	0.3
mod. Saliva natura	1.3	**2.0**	7.1	0.6	5.98	3.2	5.0	0.1	6.7	6.3	0	0

Abbreviations: DCPD (dicalcium phosphate dihydrate), OCP (octacalcium phosphate),

HA (hydroxyapatite), CaF_2 _(calcium fluoride)

### Specimen preparation and lesion formation

One hundred and fifty-five permanent bovine central and lateral incisors were obtained from newly slaughtered cattle (Figure 4a). The bulk of the adherent soft tissues was carefully removed using scalpels (disposable scalpel; Feather, Osaka, Japan), and the teeth were stored in 0.9% sodium chloride solution until further processing (Fresenius; Bad Homburg, Germany). Two enamel specimens (4 × 4 × 3 mm^³^) were prepared from the labial aspect of each crown under running tap water using a diamond-coated band saw (Exakt 300cl; EXAKT Apparatebau, Norderstedt, Germany). Two dentin specimens were prepared from the cervical regions: one specimen from the oral and one from the buccal aspect (Figure 4a). Enamel and dentin surfaces were ground flat and polished progressively up to 4,000 grit (silicon carbide abrasive paper; Hermes, Hamburg, Germany; Figure 4a). The surfaces were examined under a stereomicroscope (Axioplan 2; Carl Zeiss, Göttingen, Germany) with a magnification factor of 10 to ensure absence of any defects or physical damage. One enamel specimen from each incisor was then embedded in epoxy resin (Technovit 4071; Heraeus Kulzer, Wehrheim, Germany; Figure 4b), while the natural surface was kept free from resin. The flat parts of the specimens were ground and polished up to 4,000 grit (silicon carbide; Struers, Ballerup, Denmark), thereby removing the outer parts of the enamel (approximately 200 μm).

Artificial subsurface lesions were created in half of the enamel specimens (Figure 4b) as described previously [29]. In addition, one quarter of the surface of each specimen was covered with acid-resistant nail varnish (Jet-Set; L'Oréal, Paris, France) to serve as control for sound enamel (Figure 4b). Enamel subsurface lesions were prepared by immersion in 5 liters of a solution containing 6 μM MHDP, 3 mM CaCl_2 _× 2 H_2_O, 3 mM KH_2_PO_4_, and 50 mM CH_3_COOH (Merck, Darmstadt, Germany) at a pH of 4.95 in an incubator (37°C; BR 6000; Heraeus Kulzer, Hanau, Germany) for 19 days. The pH value was monitored daily (pH electrode GE 100 BNC, connected to pH meter GMH 3510; Greisinger, Regenstauf, Germany), and slight elevations were corrected by the addition of small amounts of HCl to maintain constant pH values between 4.92 and 4.98 during the demineralization period. Standard buffer solutions (Sigma-Aldrich, Steinheim, Germany) with nominal pH values of 4.0 and 7.0 and an accuracy of 0.01 units were used to calibrate the pH meter. All specimens (enamel, demineralized enamel, and dentin) were then sterilized with cold gas (ethylene oxide; 55°C for 30 minutes; German Heart Institute Berlin, Germany), and were vented for eight hours [30].

### Experimental phase

Two dentin and two enamel (one sound and one artificially demineralized enamel specimen) specimens were inserted into every buccal aspect of each removable denture (Figures 3a and 4d). The specimens were positioned in the regions from the second premolar to the second molar (Figure 3a). Resinated wax (Supradent; Oppermann-Schwendler, Bonn, Germany) was used to fix the specimens into manually prepared cavities at the buccal sides of the removable dentures (Figure 3a). The region not intended to be exposed to the oral environment (serving as control) was covered with acid-resistant nail varnish (Jet-Set) and flowable composite (Tetric EvoFlow; Ivoclar Vivadent, Ellwangen, Germany; Figure 3). The sound specimens' surfaces were divided into three parts: control (sound/no exposure), effect 1 (effect after five weeks of *in situ *exposure), and effect 2 (effect after seven weeks of *in situ *exposure; Figure 3c, d). The artificially demineralized enamel specimens comprised two control areas (sound and artificial demineralization; Figure 3b). After each exposure, the specimens were carefully removed and stored in saline solution (sodium chloride, 0.9%; Fresenius) until further evaluation.

### Investigation of the salivary flow rate

Unstimulated salivary flow rate of the patients was determined at each visit. All patients collected their saliva over a period of five minutes during which they were sitting upright and spat into a measuring cup, which was then weighed. The amount of saliva in grams was calculated. Previous smoking or mastication was waived, and the measurements were performed as described previously [30].

### Questionnaire

Patient characteristics such as age and sex were recorded. The patients were questioned about the frequency of saliva substitute use and the perceived persistence of the substitute in the oral cavity. The remaining amount of saliva substitute was collected and weighed to evaluate consumption. All of the questions concerning hyposalivation and quality of life were answered using the German school mark scale (1-6; 1 = very good, 6 = poor), which was familiar to the patients. Questionnaires referenced to a previous trial [16] and to surveys from the European Organization for Research and Treatment of Cancer (EORTC) QOQ-C33 and the EORTC Head and Neck (H&N35) [31]. The subjects responded to these questionnaires at six time intervals: prior to participation as well as after 5, 7, 8, 13, and 15 weeks.

### Microradiographic analysis

After *in situ *exposure, all of the specimens were mounted on transparent plexiglass microscopic slides (plexiglass-microscope slide; dia-plus, Oststeinbek, Germany) and cut into 300-μm-thick sections (perpendicular to their surfaces). These slices were ground (1,200, 2,400, 4,000 grit; Exakt) to achieve plane parallelism (Figure 4e) on wet abrasive paper (Hermes) until the remaining thickness of each slice was approximately 100 μm (± 10; Figure 4e). The width of the parallel specimens was verified using a digital micrometer with a precision of 1 μm (outside micrometer Digimatic; Mitutoyo, Kawasaki, Japan). Contact microradiographs of the enamel and dentin specimens were obtained using a nickel-filtered copper (CuKα) X-ray source (PW 1830/40; Philips, Kassel, Germany) operating at 20 kV and 10 mA for dentin as well as 20 kV and 20 mA for enamel. The exposure time for enamel and dentin was 5 seconds. During the radiographic procedures, the dentin specimens were treated with ethylene glycol (99%; Sigma-Aldrich, Munich, Germany) to avoid shrinkage [32]. An aluminum step wedge was used to generate all of the microradiographs. The radiation source-to-film distance was 34 cm. A high-resolution film (motion picture fine grain positive film 71337; Fujifilm, Tokyo, Japan) was used and developed under standardized conditions according to the manufacturer's recommendations.

The microradiographs were studied using a digital image-analyzing system (CCD video camera modul XC77E; Sony, Tokyo, Japan) interfaced with a microscope (Axioplan; Zeiss, Oberkochen, Germany) and a personal computer (ASUS P4P800X; ASUS, Taipei, Republic of China in Taiwan). Mineral loss (vol% × μm) was calculated by integrating the difference between the mineral content (vol%) in sound and demineralized enamel and dentin over the depth of the mineral lesion (μm). For all specimens, the lesion depth was defined as the distance from the surface to the location in the lesion where the mineral content was greater than 95% of the mineral content of sound enamel or dentin. The mineral volume percentage of sound enamel was set at 87% of the total volume, and that of sound dentin was set at 50% (TMR for Windows 2.0.27.2; Inspektor Research Systems, Amsterdam, The Netherlands).

If one specimen demonstrated surface erosions after *in situ *exposure, an adjacent sound part of the specimen was used for microradiography to permit adjustment of the starting point. Mineral loss and lesion depth at the specimens' surfaces were determined separately for each test field (Figure 3). Mineral losses in sound areas (ΔZ_Sound_; S in Figure 3) were subtracted from the respective values determined for artificially demineralized areas (ΔZ_Demin_; D in Figure 3b) and from the areas that were exposed to saliva substitutes for either five (ΔZ_Effect 1_) or seven weeks (ΔZ_Effect 2_) [33,34]. In artificially demineralized specimens, the values of the demineralized fields were subtracted from those obtained for the particular effect fields (E1 and E2 in Figure 3). Changes in mineral loss (ΔΔZ_Effect 1 _= ΔZ_Demin _- ΔZ_Effect 1_; ΔΔZ_Effect 2 _= ΔZ_Demin _- ΔZ_Effect 2_) were also determined. Positive and negative ΔΔZ values indicated remineralization and demineralization, respectively. Lesion depths were determined using analogous methods.

### Statistical analyses

Sample size was calculated using nQuery (version 3.0; Statistical Solutions, Cork, Ireland) prior to the start of this *in situ *study. To determine an appropriate specimen quantity to achieve an adequate power of 80% and a defined significance level of 5% (p < 0.05), the number of participants was determined to be 38 (in anticipation of a dropout rate of 10%). The expected mean value of mineral loss was 200 vol% × μm with a standard deviation of 400 vol% × μm. These values were calculated taking into account the ascertained values of previous *in vitro *studies [27,33]. In detail, those studies gave informations about the required contact time between specimens surface and saliva substitute *in vitro*, thus these results served as base for determining the specimens *in situ *contact time storage. After transversal microradiography evaluation and collection of the questionnaires, statistical analyses will be conducted using a *t*-test for paired samples. In the case of non-normally distributed values, we will apply Wilcoxon's signed-rank test. Commercially available software (PASW for Windows, version 18.0; SPSS, Munich, Germany) will be used for all statistical computing.

## Results

Hitherto, 14 patients with oral dryness who reside in Berlin have been recruited for the present study. These patients completed both test phases (Figure 1). Up to now, no reasons for early termination of the trial have been identified. Therefore, evaluation of the data will be performed after recruitment of the last participant. For this reason patient/specimen allocation will remain blinded.

## Discussion

Hyposalivation is mostly associated with various syndromes, diabetes, vitamin deficiency, menopause, salivary gland hypofunction due to inflammation, infection, the use of various drugs, or radiotherapy. Since incidence of head-and-neck cancer [35] and amount of drug revenue increases with age, primarily older patients suffer from hyposalivation [36]. Furthermore, periodontal diseases occur predominantly in elderly patients and often lead to gingival recessions [37]. Additionally, progressive attrition/abrasion during prolonged utilization of the teeth causes dentin exposure [38]. Since dentin is not as resistant as enamel to acid exposure, earlier and more severe demineralization can be expected [39]. Thus, carious lesions located at the cervix of the teeth develop easily during radiation therapy [40,41]. Therefore, in the present study, both enamel and dentin were assessed.

The teeth used in the present study were obtained from newly slaughtered cattle. One of the major disadvantages of human teeth compared to bovine teeth is the occurrence of defects such as initial carious lesions. It can be assumed that bovine teeth are rarely influenced by external factors (*e.g*., acids, fluorides), and, thus, the biological spread of bovine teeth is relatively small [25,42]. In contrast, human teeth usually exhibit an inconsistent age and source, which might result in a variable composition that leads to larger variations in the test response. Due to their similar chemical composition, general availability and large size, bovine teeth are a suitable substitute for human dental hard tissues in *in situ *examinations, and a more uniform reaction can be expected using these specimens [42]. Moreover, lesion formation in bovine enamel is very similar to the demineralization process observed in human enamel [25]. However, bovine enamel is generally considered a more porous material compared to human enamel, and, thus, bovine enamel may be susceptible to accelerated demineralization [43]. For *in situ *studies into caries prevention, sterilization of tooth specimens is essential. Sterilization of enamel and dentin using ethylene oxide has not been considered to induce relevant effects with *in situ *studies of de- and remineralization [44].

As a precursor of dental caries, initial subsurface lesions appear in many patients [1]. These lesions were simulated in the present study by the artificial demineralization (*i.e*., subsurface lesion) of half of the enamel specimens before *in situ *incorporation. Furthermore, an investigation of the remineralization processes requires the utilization of artificially demineralized enamel specimens that are adjacent to sound specimens. Due to the possibility of disintegration during the *in situ *exposure, the dentin specimens were not artificially demineralized. Four specimens that were obtained from one bovine tooth were embedded into the removable denture of one patient during each experimental phase (Figures 3a and 4). This procedure ensured that the specimens from one tooth were only used for one patient and that one bovine individual-to-one patient regimen was maintained. Furthermore, the enamel and dentin specimens correlated with respect to their source during each test phase, and this permitted a comparison of the *in situ *effects of demineralization and remineralization between dentin and enamel.

To investigate the effects of the study medication on the specimen surfaces after a wearing period of five and seven weeks, the surfaces were divided as described (Figure 3). By covering the respective surface areas with acid-resistant nail varnish and a flowable composite, the sound and artificially demineralized regions were maintained during the entire *in situ *investigation for the microradiographic analyses. Additionally, the coverage of the effect fields (E1; Figure 3) after five weeks ensured that this surface areas were not affected by additional de- and remineralizing effects. This method allowed the determination of distinguishable test areas.

Specimens attached on natural teeth could interfere with mastication and articulation, and, therefore, specimens were fixed in removable dentures. However, it has to be taken into account that wearing a dental prosthesis (full or partial) can significantly affect the composition of the resident oral microflora, and, following, the results might be influenced by this factor [45]. Therefore, this factor should be kept in mind when interpreting the upcoming data. Unlike former *in vitro *studies [46], the coverage areas were additionally fixed with flowable composite to ensure the ability to withstand mechanical forces during the study period. Up to now no visible gaps between the specimen surfaces and the coverings have been recognized. Moreover, the additional test field coverage with composite resin seems to be advantageous for protection of surface areas. So far no coverage losses could be observed.

The intention of the chosen *in situ *model was to mimic the natural caries process and provide clinically relevant information in a relatively short period of time without causing irreversible tissue changes in the panelists' dentition. Generally, clinical caries trials are limited to investigations by a dental explorer, and to using radiographs to identify and validate demineralizations at sites usually not visible directly. Thus, the caries process is determined at a relatively late stage and patients are exposed to X-rays resulting in an increasing radiation exposure. In contrast, the *in situ *model presented here offers the integration of transversal microradiography (TMR) as a basic science analytical technique. TMR offers high sensitivity and ensures waiving radiation exposure for panelists [47]. Generally, various experimental methods are available for analyzing subsurface lesions of bovine enamel and dentin specimens. These include transversal microradiography [48], polarized microscopy [49], microhardness testing [50,51], electric caries monitoring [52], transversal wavelength-independent microradiography [53], optical coherence tomography [54], and scanning electron microscopy [55,56]. With the exception of transversal microradiography (TMR), all mentioned technologies reveal some shortcomings with regard to accuracy when specimens are analyzed according to the mentioned parameters mineral loss and lesion depth. In contrast, TMR allows a direct measurement of the longitudinal mineral distribution as a profile in a subsurface lesion and has long been established and recognized as a gold standard for analyzing mineral content changes over time [57]. Consequently, TMR is considered a surrogate outcome measure that is directly impacted by the intervention. Thus, microradiographic outcome was selected as a surrogate endpoint in the present study.

Since fluorides are commonly found in foodstuff such as salt, fish, and mineral water, the study implementation required a renunciation of several foods in terms of a fluoride-free diet. This adjustment was only established for the (short) *in situ *period, and thus, might represent a lower personal burden for the patients compared with long-term clinical trials [47]. Altogether, an *in situ *model might better conform with the patients' requirements, and these always have to be weighed against the background of protracted cancer-related therapies.

Saliva substitutes such as the widespread Glandosane, which contains acids and a relatively low amount of calcium and phosphates (and, therefore, is undersaturated with respect to calcium phosphates), have demonstrated demineralizing properties *in vitro *[1,25,26,33]. From the perspective of dental medicine, neutral or even remineralizing effects of artificial salivas would seem preferable [48]. Glandosane has a pH value of 5.3, whereas the pH value of the modified SN is approximately 5.98. Due to the strict permission criteria for the modification of existing saliva substitutes (German Act of Medical Devices), a higher pH value could not be adjusted for SN. Even solutions with low pH values or a large amount of titratable acids do not cause demineralization in dental hard tissues, if an appropriate saturation is used with respect to relevant calcium phosphates via a reduction of the apatite solubility [58]. In previous *in vitro *studies, supersaturated solutions such as modified SN was observed to remineralize enamel and dentin over a period of five weeks, and this has been attributed to the addition of calcium, phosphates and fluorides [33]. Human saliva can be considered a supersaturated solution with respect to hydroxyapatite (HA), and has a mean pH value of 6.7 under physiological conditions [1]. In addition to flushing purposes, saliva also acts as a buffer. Bicarbonate and phosphate buffers in particular allow for neutralization of organic acids via diffusion through the dental plaque. The saliva of patients who suffer from hyposalivation after radiotherapy reveals a decreased pH value (*i.e*., acidic pH value), which promotes the demineralizing processes of dental hard tissues.

The primary component of enamel is HA. The solubility of a substance is characterized by its solubility product (K). For a solution such as saliva, the ion product (IP) is based mainly on the calcium, phosphate, and hydroxyl concentrations. The thermodynamic driving force of enamel de- or remineralization is a function of the degree of saturation with respect to HA (DS_HA_) [59,60], and the degree of saturation of a solution (DS) can be defined by dividing the product of the ion activities (IP) by K, and exposing this result to the number of ions in the formula unit [61]. If DS is greater than 1, the solution is supersaturated with respect to the calculated mineral phase; if DS is less than 1, the solution is undersaturated; in case of DS equals to 1, the solution is saturated, and no net dissolution or precipitation occurs. For aqueous solutions, the degree of saturation with respect to apatites [HA, octacalcium phosphate (OCP), dicalcium phosphate dihydrate (DCPD), and calcium fluoride (CaF_2_)] can be calculated, if the pH and the concentrations of certain ions are known [62]. In a previous study [27] that determined the composition of the SN used in the present one, IONPRODUCT, which was developed by Peter Shellis [62], was used. IONPRODUCT is a computer program that calculates the ionic activity of products and the degree of solution saturation with respect to biologically relevant calcium phosphates. The input values are the concentrations of the constituent ions, pH, temperature, and atmospheric pressure. A database contains the required solubility products of each of the minerals and the relevant dissociation constants. The program can be used to determine the DS at different pH values for a given calcium concentration.

Because patient acceptance of slightly acidic saliva substitutes is higher compared to products with neutral pH values, the current pH values of SN as well as of G might support patient compliance [27,33]. In the present study, an experimental period of seven weeks was selected. The present test field design refers to former *in vitro *studies exhibiting effects after an investigation of five weeks [33,48,63]. Since artificial saliva was clinically administered *ad libitum*, no maximum daily dose could be assumed. These procedures, employed in previous *in vitro *studies, simulated an extremely intense contact between saliva substitutes and specimens during an experimental time of up to five weeks, and, hitherto, these conditions could not be replicated with clinical settings and conditions. Therefore, in comparison to former *in vitro *studies a longer examination time of seven weeks was selected.

It is important to note that unlike the conditions observed *in vitro*, a total absence of saliva is rarely observed in clinical situations. Due to a variable presence of saliva, compositional changes [15], and the decreased pH values detected in the present *in situ *study, slightly altered results should be considered. Despite the omission of factors such as nutrition, previous studies have noted the development of erosions on the specimen surface even under strictly controlled *in vitro *conditions. Variability in patient diets, especially the consumption of sour drinks/food, might lead to the development of erosion. To reduce the influence of diet on mineralization, the patients were provided with nutritional counseling. In the present study, the patients were asked to restrain from the consumption of any sour foodstuffs during the test period to avoid any bias. Furthermore, panelists' were asked to abstain from any additional intake of fluorides from food or any form of fluoride-containing oral hygiene products. Therefore, advisory information and nutrition counseling regarding fluoride uptake was conducted for all patients to illustrate the need of a fluoride-free diet. In particular, the panelists' were required to abstain from fluoride-containing toothpaste; instead, fluoride-free toothpaste was administered.

To receive the patients' personal assessment concerning their well-being and usage of salivary substitutes, questionnaires were handed out as described before (Figure 2). The simplicity of the questionnaires which was enabled by referring to the German school mark scale (1-6; 1 = very good, 6 = poor), being familiar to the patients, allowed for straightforward answers to the questions and provided a fast and simple evaluation by the investigators. The evaluated personal well-being and the self-assessed intensity of dry mouth before and after the use of each saliva substitute can be compared with each other and with the collected data containing measured values of the amount of natural salivation and saliva substitutes consumption during each trial. In addition, the questionnaires provide important information on the general acceptance of the use of saliva substitutes in terms of taste and duration.

Optimal oral hygiene and regular oral screening is necessary in individuals with hyposalivation due to an increased risk for caries and periodontal diseases. Saliva substitutes should not only relieve the symptoms of oral dryness, but should also provide protection against demineralization. In addition to other *in situ *studies investigating caries formation and the protective effect of fluorides, the present study is the first clinical trial to evaluate the effects of saliva substitutes on dental hard tissues *in situ*. It is difficult to control human behavior, and, therefore, the data which will be obtained herein might differ from those generated in previous *in vitro *studies. Despite these considerations, the present study has the potential to provide clinical data that can improve the effects of salivary substitutes and their acceptance among patients. Thus, the results of the present study should be important for future treatment guidelines for patients suffering from hyposalivation.

## Competing interests

The authors declare that they have no conflict of interests. Materials and support were made independently available from the University, and, following, outcomes will not be influenced by a third party.

## Authors' contributions

PT is the Principal Investigator for the study described in the manuscript. PT, OW, ME, and AMK made significant contributions to the protocol validity, design and drafting the manuscript. PT, AMK and PM developed the statistical considerations for the trial. PT, OW, and ME participated in the study by enrolling patients and collecting data. PT and AMK revised the manuscript and all authors contributed to the scientific accuracy of the draft, and gave approval for the final version to be published.

## Pre-publication history

The pre-publication history for this paper can be accessed here:

http://www.biomedcentral.com/1472-6831/11/13/prepub

## References

[B1] Tschoppe P Wolgin M Pischon N Kielbassa AM Etiologic factors of hyposalivation and consequences for oral health Quintessence Int 2010 41 321 333 20305867

[B2] Atkinson J Ava J Salivary Gland Dysfunction: Causes, Symptoms, Treatment J Am Dent Assoc 1994 125 409 416 8176076 10.14219/jada.archive.1994.0059

[B3] Kielbassa AM Hellwig E Meyer-Lueckel H Effects of irradiation on in situ remineralization of human and bovine enamel demineralized in vitro Caries Res 2006 40 130 135 10.1159/000091059 16508270

[B4] Riley CM Day RL Central autonomic dysfunction with defective lacrimation; report of five cases Pediatrics 1949 3 468 478 18118947

[B5] Novacek G Plummer-Vinson syndrome Orphanet J Rare Dis 2006 1 36 10.1186/1750-1172-1-36 16978405 PMC1586011

[B6] Iannuzzi MC Rybicki BA Teirstein AS Sarcoidosis N Engl J Med 2007 357 2153 2165 10.1056/NEJMra071714 18032765

[B7] Guchelaar HJ Vermes A Meerwaldt JH Radiation-induced xerostomia: pathophysiology, clinical course and supportive treatment Support Care Cancer 1997 5 281 288 10.1007/s005200050075 9257424

[B8] Schiodt M Hermund NU Management of oral disease prior to radiation therapy Support Care Cancer 2002 10 40 43 10.1007/s005200100284 11777187

[B9] Kielbassa AM Hinkelbein W Hellwig E Meyer-Lueckel H Radiation-related damage to dentition Lancet Oncol 2006 7 326 335 10.1016/S1470-2045(06)70658-1 16574548

[B10] Jensen AB Hansen O Jorgensen K Bastholt L Influence of late side-effects upon daily life after radiotherapy for laryngeal and pharyngeal cancer Acta Oncol 1994 33 487 491 10.3109/02841869409083923 7917360

[B11] Harrison LB Zelefsky MJ Pfister DG Carper E Raben A Kraus DH Strong EW Rao A Thaler H Polyak T Portenoy R Detailed quality of life assessment in patients treated with primary radiotherapy for squamous cell cancer of the base of the tongue AR 1997 19 169 175 10.1002/(sici)1097-0347(199705)19:3<169::aid-hed1>3.0.co;2-0 9142514

[B12] Jellema AP Slotman BJ Doornaert P Leemans CR Langendijk JA Impact of radiation-induced xerostomia on quality of life after primary radiotherapy among patients with head and neck cancer Int J Radiat Oncol Biol Phys 2007 69 751 760 10.1016/j.ijrobp.2007.04.021 17560735

[B13] Coppes RP Vissink A Konings AW Comparison of radiosensitivity of rat parotid and submandibular glands after different radiation schedules Radiother Oncol 2002 63 321 328 10.1016/S0167-8140(02)00129-9 12142096

[B14] Funegard U Franzen L Ericson T Henriksson R Parotid saliva composition during and after irradiation of head and neck cancer Eur J Cancer B Oral Oncol 1994 30 230 233 10.1016/0964-1955(94)90002-7 7524881

[B15] Jensen SB Pedersen AM Reibel J Nauntofte B Xerostomia and hypofunction of the salivary glands in cancer therapy Support Care Cancer 2003 11 207 225 12673459 10.1007/s00520-002-0407-7

[B16] Momm F Volegova-Neher NJ Schulte-Monting J Guttenberger R [Different saliva substitutes for treatment of xerostomia following radiotherapy. A prospective crossover study] Strahlenther Onkol 2005 181 231 236 10.1007/s00066-005-1333-7 15827692

[B17] Vissink A s'Gravenmade EJ Panders AK Vermey A Petersen JK Visch LL Schaub RM A clinical comparison between commercially available mucin- and CMC- containing saliva substitutes Int J Oral Surg 1983 12 232 238 10.1016/S0300-9785(83)80048-9 6418670

[B18] Visch LL Gravenmade EJ Schaub RM Van Putten WL Vissink A A double-blind crossover trial of CMC- and mucin-containing saliva substitutes Int J Oral Maxillofac Surg 1986 15 395 400 10.1016/S0300-9785(86)80027-8 3091718

[B19] Epstein JB Stevenson-Moore P A clinical comparative trial of saliva substitutes in radiation-induced salivary gland hypofunction Spec Care Dentist 1992 12 21 23 10.1111/j.1754-4505.1992.tb00401.x 10895735

[B20] Furumoto EK Barker GJ Carter-Hanson C Barker BF Subjective and clinical evaluation of oral lubricants in xerostomic patients Spec Care Dentist 1998 18 113 118 10.1111/j.1754-4505.1998.tb00915.x 9680921

[B21] Regelink G Vissink A Reintsema H Nauta JM Efficacy of a synthetic polymer saliva substitute in reducing oral complaints of patients suffering from irradiation-induced xerostomia Quintessence Int 1998 29 383 388 9728149

[B22] Hahnel S Behr M Handel G Burgers R Saliva substitutes for the treatment of radiation-induced xerostomia-a review Support Care Cancer 2009 17 1131 1143 10.1007/s00520-009-0671-x 19495809

[B23] Urquhart D Fowler CE Review of the use of polymers in saliva substitutes for symptomatic relief of xerostomia J Clin Dent 2006 17 29 33 16898428

[B24] Smith G Smith AJ Shaw L Shaw MJ Artificial saliva substitutes and mineral dissolution J Oral Rehabil 2001 28 728 731 10.1046/j.1365-2842.2001.00803.x 11556953

[B25] Kielbassa AM Shohadai SP Schulte-Monting J Effect of saliva substitutes on mineral content of demineralized and sound dental enamel Support Care Cancer 2001 9 40 47 10.1007/s005200000148 11147142

[B26] Meyer-Lueckel H Schulte-Monting J Kielbassa AM The effect of commercially available saliva substitutes on predemineralized bovine dentin in vitro Oral Dis 2002 8 192 198 10.1034/j.1601-0825.2002.01762.x 12206400

[B27] Tschoppe P Kielbassa AM Toll R Meyer-Lueckel H [Modification of the mineralizing capacity of a saliva substitute (Saliva natura) on enamel in vitro] Laryngorhinootologie 2009 88 717 722 10.1055/s-0029-1224107 19554502

[B28] Tschoppe P Kielbassa AM Meyer-Lueckel H Evaluation of the remineralising capacities of modified saliva substitutes in vitro Arch Oral Biol 2009 54 810 816 10.1016/j.archoralbio.2009.06.004 19595293

[B29] Meyer-Lueckel H Chatzidakis AJ Kielbassa AM Effect of various calcium/phosphates ratios of carboxymethylcellulose-based saliva substitutes on mineral loss of bovine enamel in vitro J Dent 2007 35 851 857 10.1016/j.jdent.2007.08.006 17913327

[B30] Meyer-Lueckel H Bitter K Kielbassa AM Effect of a fluoridated food item on enamel in situ Caries Res 2007 41 350 357 10.1159/000104792 17713334

[B31] Rogers SN Lowe D Brown JS Vaughan ED A comparison between the University of Washington Head and Neck Disease-Specific measure and the Medical Short Form 36, EORTC QOQ-C33 and EORTC Head and Neck 35 Oral Oncol 1998 34 361 372 10.1016/S1368-8375(98)00031-1 9861341

[B32] Ruben J Arends J Shrinkage prevention of in vitro demineralized human dentine in transversal microradiography Caries Res 1993 27 262 265 10.1159/000261547 8402798

[B33] Tschoppe P Meyer-Lueckel H Kielbassa AM Effect of carboxymethylcellulose-based saliva substitutes on predemineralised dentin evaluated by microradiography Arch Oral Biol 2008 53 250 256 10.1016/j.archoralbio.2007.10.001 18070615

[B34] Kawasaki K Ruben J Tsuda H Huysmans MC Takagi O Relationship between mineral distributions in dentine lesions and subsequent remineralization in vitro Caries Res 2000 34 395 403 10.1159/000016614 11014906

[B35] DePinho RA The age of cancer Nature 2000 408 248 254 10.1038/35041694 11089982

[B36] Gueiros LA Soares MS Leao JC Impact of ageing and drug consumption on oral health Gerodontology 2009 26 297 301 10.1111/j.1741-2358.2009.00284.x 19392837

[B37] Albandar JM Kingman A Gingival recession, gingival bleeding, and dental calculus in adults 30 years of age and older in the United States, 1988-1994 J Periodontol 1999 70 30 43 10.1902/jop.1999.70.1.30 10052768

[B38] Van't Spijker A Rodriguez JM Kreulen CM Bronkhorst EM Bartlett DW Creugers NH Prevalence of tooth wear in adults Int J Prosthodont 2009 22 35 42 19260425

[B39] Saunders RH Jr Meyerowitz C Dental caries in older adults Dent Clin North Am 2005 49 293 308 10.1016/j.cden.2004.10.004 15755406

[B40] Kielbassa AM Wrbas KT Dornfeld B Hellwig E Schade-Brittinger C [*In vitro *and *in situ *studies on the effects of tumor radiotherapy on the developement of caries in human dentin] Dtsch Zahnarztl Z 1999 54 31 37

[B41] Kielbassa AM In situ induced demineralization in irradiated and non-irradiated human dentin Eur J Oral Sci 2000 108 214 221 10.1034/j.1600-0722.2000.108003214.x 10872992

[B42] Lynch RJ Duckworth RM Model parameters and their influence on the outcome of in vitro demineralisation and remineralisation studies Monogr Oral Sci 2006 19 1 Basel: Karger 65 85 16374029 10.1159/000090586

[B43] Amaechi BT Higham SM Edgar WM Factors influencing the development of dental erosion in vitro: enamel type, temperature and exposure time J Oral Rehabil 1999 26 624 630 10.1046/j.1365-2842.1999.00433.x 10447814

[B44] Thomas RZ Ruben JL ten Bosch JJ Huysmans MC Effect of ethylene oxide sterilization on enamel and dentin demineralization in vitro J Dent 2007 35 547 551 10.1016/j.jdent.2007.03.002 17475389

[B45] Marsh PD Percival RS The oral microflora--friend or foe? Can we decide? Int Dent J 2006 56 233 239 16972398 10.1111/j.1875-595x.2006.tb00107.x

[B46] Tschoppe P Zandim DL Sampaio JE Kielbassa AM Saliva substitute in combination with high-concentrated fluoride toothpaste. Effects on demineralised dentin in vitro J Dent 2010 38 207 213 10.1016/j.jdent.2009.10.005 19861146

[B47] Zero DT In situ caries models Adv Dent Res 1995 9 214 231 8615944 10.1177/08959374950090030501

[B48] Tschoppe P Meyer-Lueckel H Toll R Kielbassa AM [In vitro analysis of a new saliva substitute (Saliva natura) on enamel and dentin] Laryngorhinootologie 2007 86 723 727 10.1055/s-2007-966495 17487817

[B49] Crabb HS Darling AI X-ray absorption studies of human dental enamel Oral Surg Oral Med Oral Pathol 1956 9 995 1009 10.1016/0030-4220(56)90303-6 13370088

[B50] Buchalla W Imfeld T Attin T Swain MV Schmidlin PR Relationship between nanohardness and mineral content of artificial carious enamel lesions Caries Res 2008 42 157 163 10.1159/000128559 18446023

[B51] Kielbassa AM Wrbas KT Schulte-Monting J Hellwig E Correlation of transversal microradiography and microhardness on in situ-induced demineralization in irradiated and nonirradiated human dental enamel Arch Oral Biol 1999 44 243 251 10.1016/S0003-9969(98)00123-X 10217515

[B52] Petersson LG Kambara M Remineralisation study of artificial root caries lesions after fluoride treatment. An in vitro study using electric caries monitor and transversal micro-radiography Gerodontology 2004 21 85 92 10.1111/j.1741-2358.2004.00017.x 15185988

[B53] Thomas RZ Ruben JL de Vries J ten Bosch JJ Huysmans MC Transversal wavelength-independent microradiography, a method for monitoring caries lesions over time, validated with transversal microradiography Caries Res 2006 40 281 291 10.1159/000093186 16741358

[B54] Holtzman JS Osann K Pharar J Lee K Ahn YC Tucker T Sabet S Chen Z Gukasyan R Wilder-Smith P Ability of optical coherence tomography to detect caries beneath commonly used dental sealants Lasers Surg Med 2010 42 752 759 10.1002/lsm.20963 20848554 PMC3369270

[B55] Kielbassa AM Pioch T Rowbotham F Hellwig E In vivo demineralization of irradiated human enamel. A SEM study Acta Med Dent Helv 1997 2 193 198

[B56] Kielbassa AM Schaller H-G Hellwig E [Qualitative observations of in situ caries in irridiated dentin. A combined SEM and TMR study] Acta Med Dent Helv 1998 3 161 168

[B57] Damen JJ Exterkate RA ten Cate JM Reproducibility of TMR for the determination of longitudinal mineral changes in dental hard tissues Adv Dent Res 1997 11 415 419 10.1177/08959374970110040601 9470498

[B58] Aoba T Solubility properties of human tooth mineral and pathogenesis of dental caries Oral Dis 2004 10 249 257 10.1111/j.1601-0825.2004.01030.x 15315640

[B59] van Dijk JW Borggreven JM Driessens FC Chemical and mathematical simulation of caries Caries Res 1979 13 169 180 10.1159/000260398 286645

[B60] Gao XJ Elliott JC Anderson P Scanning microradiographic study of the kinetics of subsurface demineralization in tooth sections under constant-composition and small constant-volume conditions J Dent Res 1993 72 923 930 10.1177/00220345930720051401 8501290

[B61] Anderson P Hector MP Rampersad MA Critical pH in resting and stimulated whole saliva in groups of children and adults Int J Paediatr Dent 2001 11 266 273 10.1046/j.1365-263X.2001.00293.x 11570442

[B62] Shellis RP A microcomputer program to evaluate the saturation of complex solutions with respect to biominerals Comput Appl Biosci 1988 4 373 379 3416199 10.1093/bioinformatics/4.3.373

[B63] Meyer-Lueckel H Tschoppe P Hopfenmuller W Stenzel WR Kielbassa AM Effect of polymers used in saliva substitutes on demineralized bovine enamel and dentin Am J Dent 2006 19 308 312 17073209

